# Urinary metabolite profiles differ by tumor size and malignancy in dogs with mammary tumors

**DOI:** 10.3389/fvets.2026.1834546

**Published:** 2026-07-14

**Authors:** Robin Moore, Alessandra Estrela-Lima, Soile Turunen, Sarah Victoria Holm, Marko Lehtonen, Olli Kärkkäinen, Anna Hielm-Björkman

**Affiliations:** 1 Department of Equine and Small Animal Medicine, University of Helsinki, Helsinki, Finland; 2 Department of Veterinary Anatomy, Pathology and Clinics, School of Veterinary Medicine and Zootechnics, Federal University of Bahia, Bahia, Brazil; 3 School of Pharmacy, University of Eastern Finland, Kuopio, Finland

**Keywords:** cancer, canine, mammary, metabolite profiles, metabolomics, urine

## Abstract

**Background:**

Urine metabolomics may offer a non-invasive way to detect systemic metabolic alterations associated with canine mammary tumors, but previous studies have been few and have not accounted for diet as a potential confounder.

**Methods:**

In this observational case–control study, urine from 118 client-owned female dogs in Finland was analyzed by untargeted liquid chromatography-mass spectrometry, including 71 dogs with mammary tumors and 47 tumor-free controls. Case dogs were further evaluated by tumor malignancy (28 benign, 27 malignant, 16 with both benign and malignant tumors), tumor size (53 small, 12 medium, 6 large), and tumor number (43 single, 28 multiple). Statistical models were adjusted for diet, age, body size, and sterilization status.

**Results:**

After covariate adjustment, no features remained significant in the overall case–control comparison or in analyses of tumor number, whereas tumor size showed the clearest signal. Three features remained significant in the covariate-adjusted analysis of variance for tumor size, and per-contrast linear models identified four false discovery rate-significant urinary features associated with tumor size and four associated with tumor malignancy. All showed lower intensities in tumor-bearing dogs than in controls. Hippurate was the most robust and consistently supported finding, with lower levels in dogs with large tumors. Several unknown aromatic and sulfur-containing features covaried with hippurate and tumor size, suggesting a broader host metabolic response. Exploratory pathway analysis indicated glucose homeostasis as the strongest enrichment signal in adjusted models, although this did not remain significant after false discovery rate correction. Adjustment for diet and other background variables substantially reduced the number of significant findings, highlighting the importance of accounting for physiological and environmental variation in veterinary metabolomics.

**Conclusion:**

Urine metabolomics detected a limited but biologically plausible metabolic signature associated primarily with tumor size rather than with overall case–control status or tumor number. Hippurate and a small set of unknown features emerged as candidate urinary markers of tumor-associated systemic metabolic change, supporting the value of urine as a non-invasive matrix for studying canine mammary tumors while underscoring the need for careful covariate control.

## 1 Introduction

Canine mammary tumors (CMTs) are among the most common neoplasms in female dogs, particularly in populations where elective neutering is less common, and approximately half are malignant (1–3). Histopathology remains the diagnostic standard (3), and early detection directly influences prognosis (4). Metabolomics is increasingly used in the analysis of canine biosamples (5), and urine metabolomics has recently been applied to mammary cancer in dogs (6, 7). Several candidate urinary biomarkers have been suggested, including altered tryptophan and tyrosine metabolites (7). Although metabolomics studies on CMTs remain scarce, work in human breast cancer (BC) suggests that metabolomics may be useful for characterizing disease-associated metabolic changes (8). Comparable alterations in tryptophan metabolites across BC and CMT studies may indicate partly shared pathogenic pathways (9).

CMTs and human BC share several clinically relevant features, including spontaneous development, comparable histopathological frameworks, and similarities in aspects of tumor morphology, molecular biology, and metastatic behavior (7, 10–15). Dogs may therefore offer value as a comparative model in some areas of breast cancer research, while the differences between species remain important and should not be overlooked (16–18). In particular, previous studies have identified both shared genomic features, such as BRCA1/2-related findings, and species-specific differences, including the apparent lack of HER2-enriched tumor genotypes in dogs (17, 18). In addition, companion dogs share environmental and lifestyle factors that may be relevant to cancer development and progression in humans (9).

To our knowledge, six studies have specifically investigated canine mammary tumor metabolomics (6, 7, 19–22), of which two focused on urine metabolite profiles. Of these two urine studies, one was a targeted LC-based analysis of 12 tyrosine- and tryptophan-related metabolites, whereas the other used NMR and did not report differences in metabolite concentrations between healthy dogs and dogs with malignant or benign tumors. Notably, none of the previous articles considered diet. This is a relevant methodological gap, as canine diet is known to influence metabolic profiles (23, 24). In Finland, dogs primarily consume ultra-processed dry foods (kibble diets, KDs), raw meat-based diets (RMBDs), ultra-processed wet foods (canned, pouches, rolls) or mixed home-cooked meals, including leftovers from the owner’s meals (25), often in combination. In our previous work, we observed associations between diet type and both health outcomes (25–27) and metabolic profiles (23) when comparing dogs fed commercially available RMBDs and KDs. These findings suggest that diet may act as an important background variable in urine metabolomics studies of CMTs and should be considered when interpreting disease-associated metabolic variation.

We therefore aimed to investigate whether urinary metabolites could distinguish dogs with mammary tumors from controls, differentiate between benign and malignant tumors, and identify metabolic variation associated with tumor growth characteristics and subtype. To improve interpretability and clinical relevance, dietary factors were incorporated as covariates.

## 2 Materials and methods

### 2.1 Clinical study design and setting

We conducted an observational, case–control study comparing home-living adult female companion dogs with mammary tumors (cases) to tumor-free control dogs in Finland. Dog size, age, and spay status were not matched between cases and controls and were instead included as covariates in all statistical models (statistical methods reported separately).

#### 2.1.1 Animals: eligibility, recruitment, and groups

The study group comprised client-owned adult female dogs diagnosed with mammary tumors, recruited from various collaborating veterinary clinics in southern Finland. Control dogs were recruited at veterinary examination and dog-owner events in southern and eastern Finland. All dogs recruited as controls were evaluated by a veterinarian by mammary gland palpation at enrollment and were included as tumor-free controls only if no palpable mammary masses were detected and the owner reported no previous diagnosis of mammary tumors. Dogs in which mammary masses were detected by palpation were not included as controls; a subset of these dogs was referred for further clinical evaluation and, when subsequently submitted to surgical excision and histopathology, contributed to the case group if inclusion criteria were met. Both groups included dogs of diverse breeds and sizes, all of which were maintained in typical domestic environments.

##### 2.1.1.1 Owner questionnaires and diet ascertainment

Owners completed two questionnaires covering background and lifestyle factors (including diet) and, for cases, details of the tumor diagnosis. The first food-frequency questionnaire (FFQ1) asked owners to classify diet using four categories—Dry Processed (kibble), Raw, Home-cooked, and/or Wet Processed—and to report the dog’s meal on the evening before urine sampling and whether that meal reflected the habitual adult diet (response rate 92.6%). Because relative proportions were not collected in this instrument, only clearly “pure” diets could be unambiguously assigned from FFQ1. To obtain quantitative composition, a second retrospective questionnaire asked owners to allocate the dog’s diet across the same four categories in 10% increments, to confirm that the components summed to 100%, and to indicate whether the reported mix reflected the habitual diet during the 6 months before urine sampling (response rate 48%). Information from both questionnaires was harmonized to derive a semi-quantitative daily diet profile for each dog.

##### 2.1.1.2 Diet covariates used in modeling

Using the harmonized diet information, each dog was assigned to one of nine mutually exclusive diet categories: (1) Only kibble (100% KD); (2) Only kibble + wet processed; (3) Mostly processed (i.e., either kibble or wet-processed) with some home-cooked; (4) Mostly processed (i.e., either kibble or wet-processed) with some raw; (5) Mostly home-cooked, no raw; (6) Approximately half raw and half kibble; (7) Mostly raw (predominantly RMBD); (8) “Everything” (mixed across types); and (9) Unknown. These nine categories were encoded as a single categorical covariate and included in all statistical models alongside dog size, age and sterilization status. We did not conduct separate diet-stratified analyses; diet information was used solely as covariates to adjust associations between case–control status and metabolomic outcomes. Details of model parameterization are provided in the Statistical Analysis section.

#### 2.1.2 Urine sample collection and surgical removal of mammary tumors

Owners collected free-catch morning urine from overnight-fasted dogs into sterile cups supplied in advance. Samples were kept refrigerated at approximately +4 °C before and during same-day transport to the clinic, transferred to 50-mL sterile tubes, and stored at −80 °C until analysis. The exact time from collection to freezing was not systematically recorded. Samples underwent two thawing events before liquid chromatography tandem mass spectrometry (LC–MS/MS) analysis: first during aliquoting and sample preparation, and again before injection into the LC–MS/MS system. For case dogs, urine was collected on the morning before anesthesia, tumor excision, and any ovariectomy performed at the same visit. Hence in the present study, sterilized dogs were defined as those that had undergone ovariectomy before the study visit. Dogs classified as in estrus were in estrus at the time of sample collection. Estrus status was determined by a veterinarian at the time of urine collection, and the owner was also asked in the food frequency questionnaire whether the dog was in estrus during sampling. Details of the anesthetic protocol used for surgery are provided in Supplementary material S4.1 but were not expected to influence urine chemistry.

#### 2.1.3 Histopathological analysis of tumors

Post-excision, mammary tissue specimens were submitted to the University of Helsinki’s veterinary pathology service for routine histopathology, and a structured report was issued for each dog. Tumor size was preferentially taken from the pathology report, reflecting routine clinical estimation at or around the time of excision. All owners were also asked to report approximate tumor size in the questionnaire, and this value was used only when tumor size was not documented in the pathology report. Tumor size was categorized as small (<3 cm), medium (3–5 cm), or large (>5 cm). Tumors were classified according to Goldschmidt et al. (28). Benign tumors were classified into three categories: benign mixed tumor (BMT), complex adenoma (CA), and simple adenoma/myoepithelioma (SA/MYO). Malignant tumors were classified into six categories: complex carcinoma (CC), tubular carcinoma (TC), ductal carcinoma (DC), adenosquamous carcinoma (ASC), invasive micropapillary carcinoma (IMC), and other malignant tumors (OMT). Benign/malignant grouping was based on the histopathological diagnosis reported for each lesion. Dogs with only benign tumors were assigned to the benign group, dogs with only malignant tumors were assigned to the malignant group, and dogs with both benign and malignant tumors were retained as a separate benign-and-malignant group. For descriptive summaries requiring one representative histological category per dog, the highest-risk histopathological diagnosis available in the report was selected, prioritizing malignant over benign lesions and among malignant lesions, diagnoses associated with more aggressive biological behavior over less aggressive diagnoses, guided by established canine mammary tumor classification and prognostic literature (4, 28). One author (AE-L), an expert in CMT pathology, reviewed the available diagnostic report information and supported interpretation of report terminology, tumor subtype, and invasive status when clarification was needed. Histology slides and paraffin blocks were not available for standardized retrospective review; therefore, histological grade could not be added consistently, and the Nottingham Prognostic Index could not be applied. A summary of tumor characteristics for all case dogs is provided in Supplementary File 1, including the dog-level benign/malignant grouping used in the analyses and the histological category used for descriptive summaries.

### 2.2 Analytical procedure

#### 2.2.1 Sample preparation

Untargeted urine metabolomics was used to compare prespecified groups (tumor-bearing vs. tumor-free animals). All samples were processed in a single analytical batch with embedded pooled quality-control (QC) samples to monitor instrument stability and analytical drift. Frozen urine was thawed on ice and prepared by protein precipitation followed by filtration prior to LC–MS/MS analysis. QC material was generated by pooling small aliquots from each study sample and was interleaved regularly throughout the run to track analytical performance. Full reagent compositions, volumes, temperatures, and centrifugation conditions are provided in Supplementary methods S4.2.

#### 2.2.2 LC–MS measurement

Samples were analyzed on a Vanquish Flex ultra-high-performance liquid chromatography (UHPLC) system coupled to a Q Exactive Focus high-resolution Orbitrap mass spectrometer (Thermo Scientific, Bremen, Germany). Complementary reversed-phase (RP) and hydrophilic interaction chromatography (HILIC) separations were performed using a Zorbax Eclipse XDB C18 column (2.1 × 100 mm, 1.8 μm; Agilent Technologies, Palo Alto, CA, United States) and an Acquity UPLC BEH Amide column (2.1 × 100 mm, 1.7 μm; Waters, Milford, MA, United States), respectively. Electrospray ionization was applied in both positive and negative modes, and data-dependent MS/MS was acquired to aid annotation. Injection order was randomized, and pooled QC samples were placed at the start and end of the sequence and at regular intervals to monitor temporal drift. Detailed chromatographic gradients, ion-source conditions, mass ranges, resolutions, and MS/MS settings are reported in Supplementary methods S4.3.

#### 2.2.3 Data collection and preprocessing

Raw data were converted and processed in MS-DIAL for peak detection and alignment using parameters adapted from the NOTAME workflow (29). Putative identification leveraged an in-house LC–MS/MS spectral library in combination with public databases and confidence was assigned according to Sumner et al. identification levels 1 (metabolite identified) to 4 (unknown) (30). Parameters for tolerances, adduct handling, alignment, feature filtering, and library matching, as well as data scope metrics, are detailed in Supplementary methods S4.4. Drift correction and missing-value imputation (random forest) were performed within the NOTAME R framework separately for each analytical mode.

#### 2.2.4 Computational MS/MS annotation of unknown features

To aid in the biological interpretation of unknown LC–MS features, we performed further formula and structure annotation with SIRIUS 6 (v6.3.3). For each feature with MS/MS data, SIRIUS computed the most likely molecular formula from isotope-pattern and fragmentation-tree analysis, followed by CSI: FingerID structure searching against curated public databases. Searches were run per ionization mode with mode-appropriate common adducts and fragments. Mass tolerances were set to ±5 ppm (MS^2^). For reporting, we retained the top-ranked candidate per feature and harmonized identification confidence to metabolomics standards. Candidates emphasized in the Results were manually checked for adduct consistency, diagnostic neutral losses/fragments and chromatographic plausibility with the separation used (RP vs. HILIC) and ionization mode. A table detailing SIRIUS parameters is included as Supplementary File 5.

#### 2.2.5 Creatinine adjustment with NMR

To account for urine dilution, LC–MS features were normalized to creatinine quantified by NMR using an internal reference compound. Agreement between NMR-quantified creatinine and its LC–MS signal was verified and is summarized in Supplementary Figure S1.

#### 2.2.6 Statistical analysis

Statistical analyses were performed in R (version 4.5.1) using the package suite MetaboAnalystR (31), and packages limma, sandwich, lmtest, robustbase, logistf, ggplot2, ggpubr, ggrepel, pheatmap, dplyr, tidyr, tibble, readr, purrr, broom, brglm2, and MASS. All statistical analysis scripts are available on GitHub.^1^

Before statistical modeling, raw LC–MS data were processed in MS-DIAL for peak detection and alignment, followed by NOTAME-based drift correction and random-forest imputation of missing values separately for each analytical mode, as described above and in Supplementary methods S4.4. Peak areas were normalized to NMR-quantified creatinine to account for urine dilution. The resulting feature table and metadata were then imported into MetaboAnalystR, where remaining missing values were imputed using variable-wise k-nearest neighbors (knn_var), followed by log₁₀ transformation using the LogNorm procedure.

For downstream analyses, feature-wise linear models were fitted as log10(intensity) ~ x + age + size + sterilization status + diet category, where x was one of the primary research variables: case–control status, tumor malignancy, tumor size, or tumor number. All models were additive and adjusted for age group (young, mid-aged, old), size class (small, medium, large), sterilization status, and diet group. Covariates were included to reduce confounding; their coefficients were not interpreted, and no interaction terms were fitted because of limited support in small strata. *p*-values were adjusted across features using the Benjamini–Hochberg false-discovery-rate (FDR) procedure. Heteroskedasticity-robust HC3 standard errors (HC3 SEs) were used for all feature-wise linear comparisons, including both control-referenced contrasts and additional pairwise contrasts among non-control disease groups. Because residual variance can differ across animals and covariate strata, HC3 SEs were used for all regression coefficients, as HC3 is recommended for small to moderate samples and mitigates the influence of high-leverage observations without altering point estimates (32). For each primary model, a covariate-adjusted analysis of variance (ANOVA) screen with Benjamini–Hochberg FDR correction was also performed in MetaboAnalystR.

After fitting the adjusted linear models, residual normality was assessed feature-wise using the Shapiro–Wilk test, with Benjamini–Hochberg FDR correction across aligned features. Visual assessment included histograms and empirical cumulative distribution plots of Shapiro–Wilk *p*-values and q-values, together with residual Q–Q plots and residual density plots (Supplementary Figures S3a–c). As sensitivity analyses, adjusted-significant features were re-analyzed using robust regression and Freedman–Lane permutation testing with 1,000 permutations. Influence diagnostics included strict leverage and Cook’s distance screens for the adjusted linear models, together with sample-level summaries of the proportion of feature-wise models in which each dog exceeded the Cook’s distance threshold.

Adjusted-significant features from the HC3-based linear models were additionally summarized in supplementary analyses using control-referenced logistic regression, with odds ratios and 95% confidence intervals estimated per 1 standard deviation increase in log₁₀ intensity; when standard maximum-likelihood estimation was unstable or separation was encountered, penalized or bias-reduced logistic fitting was used, following previously described approaches for separation and bias reduction (33, 34).

Because cases and controls differed in baseline characteristics, we additionally evaluated case–control covariate balance using standardized mean differences and covariate-overlap summaries for the core model covariates: diet group, age group, sterilization status, and dog size class. As a sensitivity analysis, the adjusted case–control model was repeated within the age-overlap subset after excluding the non-overlapping young-control stratum. Propensity-score overlap and inverse-probability-of-treatment weights were evaluated to assess the feasibility of propensity-based balancing. Furthermore, as the large-tumor group was small, tumor-size findings involving the large-tumor group were further assessed using a leave-one-out sensitivity analysis in which each large-tumor dog was omitted in turn and the control-versus-large tumor contrast was re-estimated for the key tumor-size-associated features.

#### 2.2.7 Metabolite set enrichment analysis

Features of interest from the models were used for metabolite set enrichment analysis (MSEA) in MetaboAnalyst based on RaMP-DB (Relational Database of Metabolomic Pathways) to identify metabolic pathways potentially relevant to the research questions (31, 35). MSEA used level 1 and 2 annotated features with nominal raw *p* < 0.05 from the covariate-adjusted per-contrast linear models; the input set was therefore not restricted to FDR-significant features. Common mammalian metabolic pathways were explored using either *H. sapiens* as the reference mammal, or *C. lupus familiaris* whenever available.

## 3 Results

### 3.1 Study group and data overview

**Figure 1:**
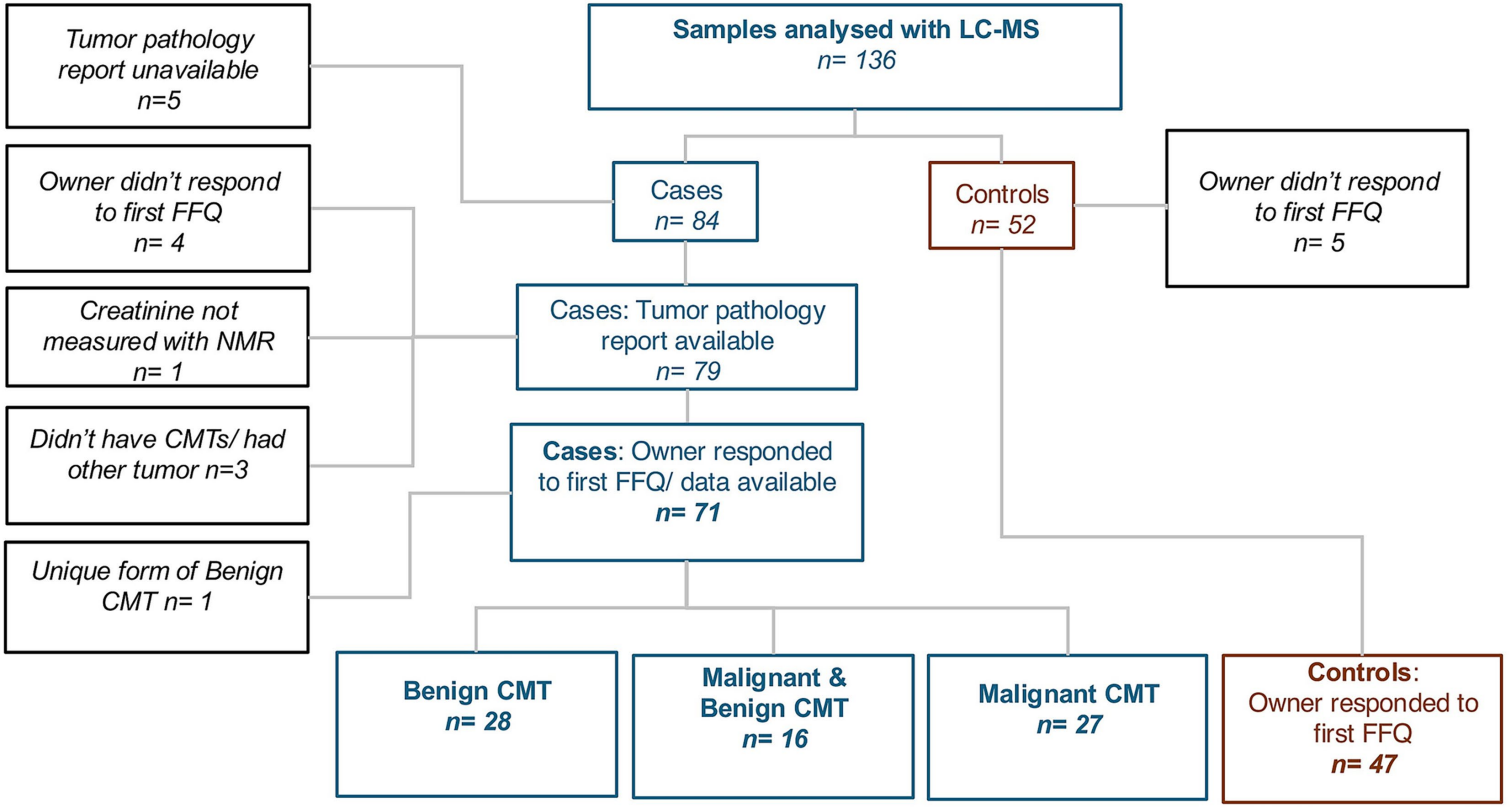
A flowchart of the experimental steps.

A flowchart of the study design, including dogs, group counts, samples analyzed, and exclusion criteria, is presented in Figure 1. An overview of the dog background characteristics, including age, sterilization status, size class, and diet category, is shown in Table 1. Dog groups corresponding to the primary outcome variables—case/control status, tumor malignancy (benign, malignant, or both benign and malignant tumors), tumor size (small, medium, large), and tumor number (single, multiple)—are presented in Tables 2, 3. Because the tumor-size and tumor-number groupings include dogs with differing malignancy status, these groupings are shown separately in Table 3.

**Table 1:** An overview of dog background characteristics.

Variable		Cases (*n* = 71)	Controls (*n* = 47)	Total (*n* = 118)	*p*-value (case vs control)
Breeds (FCI)	F0 mixed breed	18	3	21	1.20E-03*
	F1 sheepdogs and Cattledogs	6	7	13	2.00E-01
	F2 pinscher and schnauzer—molossoid	7	8	15	1.80E-01
	F3 Terriers	10	3	13	5.70E-02
	F4 Dachshunds	7	0	7	1.10E-03*
	F5 Spitz/primitive	1	3	4	7.00E-01
	F6 scent hounds	1	1	2	1.00E+00
	F7 pointing dogs	1	2	3	4.40E-01
	F8 retrievers and water dogs	5	16	21	9.70E-01
	F9 companion and toy dogs	18	1	19	2.40E-05**
	F10 sighthounds	0	4	4	9.80E-01
Age, years (Mean ± SD)†		9.49, 2.14	5.53, 3.53	7.44, 2.87	2.01E-9***
Age	Young (1–3 y.o)	0	18	18	2.67E-10***
	Mid-aged (3–8 y.o)	16	15	31	
	Old (>8 y.o)	55	14	69	
Size	Small (<10 kg)	34	7	41	3.66E-05**
	Medium (10-20 kg)	28	17	45	3.10E-01
	Large (>20 kg)	9	24	31	5.90E-03*
Spay status (intact/spayed)		45/26	29/18	74/44	0.93
Estrus (No/Yes)‡		69/2	45/2	114/4	0.67
Diets	Only kibble (100% KD)	9	20	29	9.80E-02
	Only kibble + wet processed	14	1	15	2.80E-04*
	Mostly processed with some home-cooked	21	2	23	4.05E-05**
	Mostly processed with some RMBD	8	11	19	5.70E-01
	Mostly home-cooked, no RMBD	5	0	5	8.40E-03*
	Approximately half RMBD and half KD	4	8	12	2.70E-01
	Mostly raw (predominantly RMBD)	5	2	7	2.40E-01
	Everything (mixed across types)	1	3	4	3.00E-01
	Unknown	4	0	4	2.20E-02*

KD, Ultra-processed kibble diet, RMBD, Raw meat-based diet FCI, Fédération Cynologique Internationale *p < 0.05, **p < 0.01, ***p < 0.001. †Continuous age is shown as mean ± SD and was compared using Welch’s two-sample *t*-test. Age group and other categorical variables were compared using Fisher’s exact test. ‡Estrus status refers only to intact dogs in estrus at urine collection.

**Table 2:** Overview of primary outcome variables.

Case/control	Case (*n* = 71)			Control (*n* = 47)
Tumor malignancy	Malignant (*n* = 27)	Benign (*n* = 28)	Malignant and Benign (*n* = 16)	Control (*n* = 47)
No. of tumors (single/multiple)	19 single/7 multiple	23 single/5 multiple	0 single/16 multiple	NA/NA
Size of tumors (small: < 3 cm/medium: 3–5 cm/Large: > 5 cm)	16/7/4	25/1/2	12/4/0	0/0/0

NA, not applicable.

**Table 3:** The subject groups based on tumor size and number of tumors when used as the primary outcome variable.

Size of tumors	Case (*n* = 71)
Small: < 3 cm	53
Medium: 3–5 cm	12
Large: > 5 cm	6
Number of tumors	Case (*n* = 71)
Single	43
Multiple	28

### 3.2 Distributional diagnostics, robustness, and sensitivity analyses

After alignment, 3,925 metabolite features were included in the models. A substantial proportion of urine metabolite features showed evidence of non-normal residuals after false-discovery-rate correction (*q* < 0.05): Model 1 (tumor malignancy), 1,922/3,925 features (49.0%); Model 2 (case–control), 1,945/3,925 (49.6%); Model 3 (tumor size), 1,864/3,925 (47.5%); and Model 4 (tumor number), 1,864/3,925 (47.5%). However, the main conclusions were largely unchanged in sensitivity analyses using HC3 standard errors, robust regression, and Freedman–Lane permutation testing. An overview of FDR-significant versus nominally significant findings across Models 1–4 is provided in Supplementary Table S1, and full results tables are available in Supplementary File 2.

Case–control balance diagnostics confirmed substantial baseline imbalance between cases and controls. The largest standardized mean differences were observed for exact age, age group, dog size class, and selected diet categories. Covariate-overlap checks identified non-overlap for the young age group and for two diet categories. In the age-overlap restricted case–control sensitivity analysis, the adjusted case–control comparison remained null, with no features significant after false discovery rate correction among 3,925 tested features. Propensity-score diagnostics indicated limited overlap and potentially unstable inverse-probability weighting, including 16 dogs with propensity scores <0.01 or >0.99 and one inverse-probability weight >10; therefore, propensity-weighted results were not used as a primary robustness analysis. Full balance diagnostics and sensitivity results are provided in Supplementary File 6.

### 3.3 Inclusion of covariates

We first examined correlations among study variables to guide covariate selection and avoid collinearity in multivariable models (supplementary Figure S2a). Variables capturing tumor size, histopathological subtype, tumor number (single/multiple), and case/control status were strongly intercorrelated and were therefore treated as primary metadata in separate models. Covariates were excluded if they correlated with another variable at ∣r∣ > 0.5, following the protocol guidelines (31), and the remaining covariates (sterilization status, diet, age, dog size) are shown in supplementary Figure S2b.

### 3.4 Primary differential analysis

**Figure 2:**
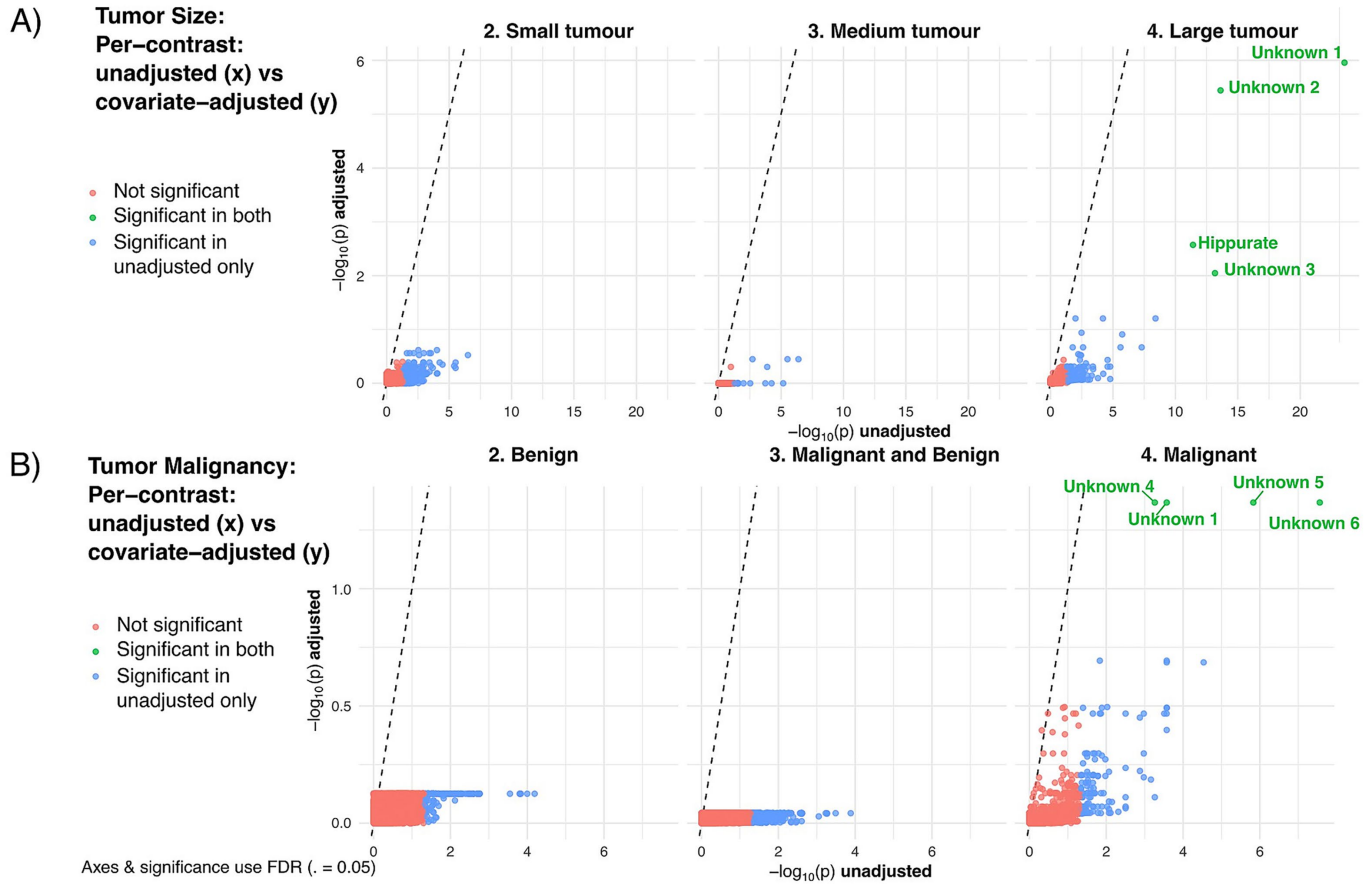
Effect of covariate adjustment on feature-level significance in the per-contrast linear models. **(A)** Tumor-size model. **(B)** Tumor-malignancy model. Each point represents one metabolite feature, plotted by its unadjusted *p*-value (x-axis) and covariate-adjusted p-value (y-axis). Green points indicate features that remained significant after false discovery rate (FDR) correction following covariate adjustment, whereas blue points indicate features that were significant before but not after covariate adjustment.

**Figure 3:**
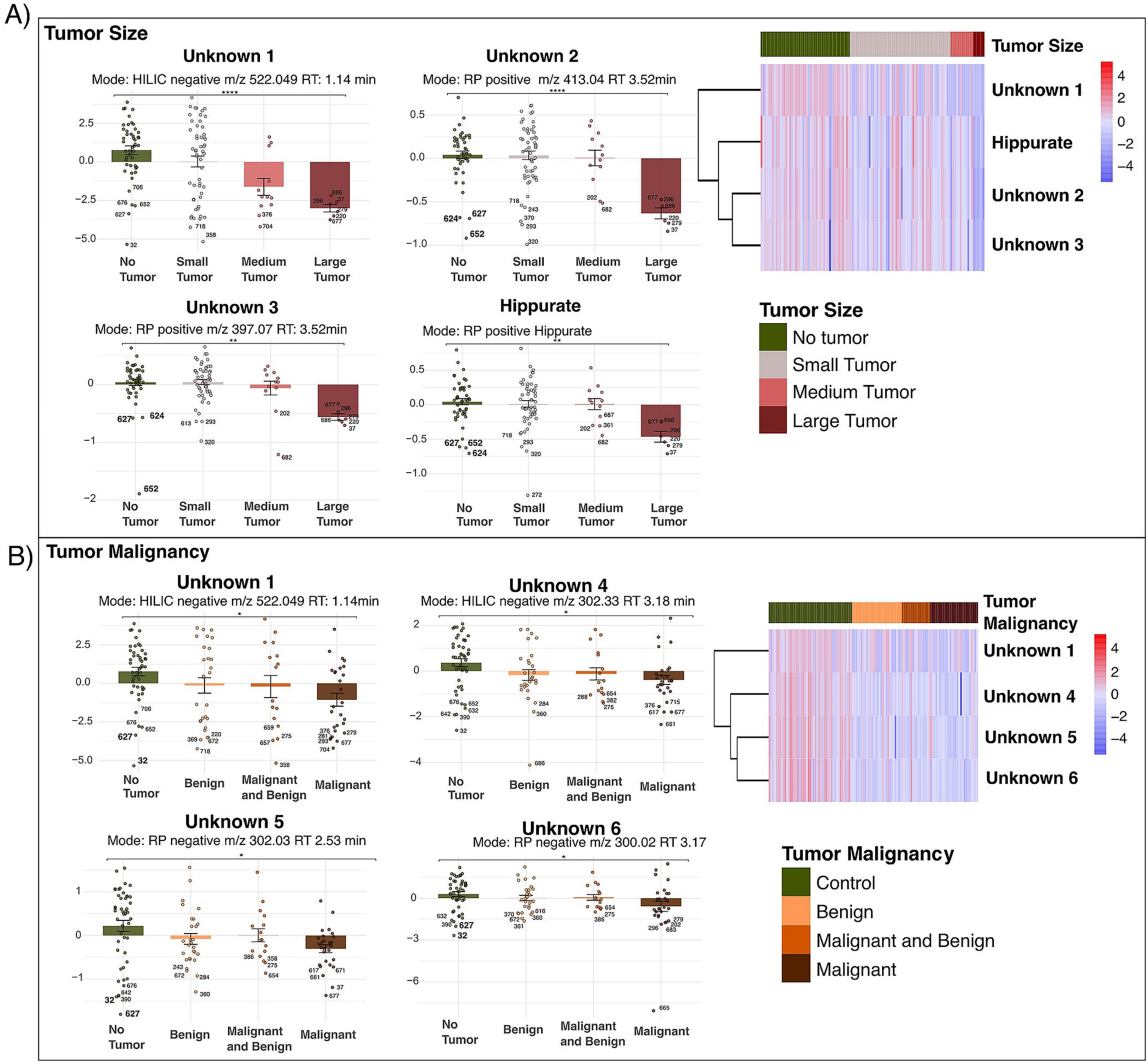
Bar plots of urine metabolite features significant in the covariate-adjusted per-contrast linear models. **(A)** Mean log10 intensity of the four FDR-significant features identified in the tumor-size analysis. **(B)** Mean log10 intensity of the four FDR-significant features identified in the tumor-malignancy analysis. Dogs in the lowest decile for each feature are annotated by ID number; bolded IDs denote control dogs. Corresponding heatmaps are shown adjacent to the bar plots, and group colors are matched across both display formats.

Of the 3,925 urine metabolite features included in the covariate-adjusted linear models, none remained significant at FDR < 0.05 in the case–control comparison or in ANOVA models evaluating tumor malignancy or tumor number, whereas three features remained significant in the ANOVA of the tumor-size model (Supplementary Table S2). Because the ANOVA tested a global tumor-size effect across groups, whereas the per-contrast models tested specific group contrasts, the significant feature sets were not expected to be identical. The per-contrast linear models identified four FDR-significant features associated with tumor malignancy and four FDR-significant features associated with tumor size after covariate adjustment (FDR < 0.05; Figures 2A,B). For all of these features, metabolite intensities were lower in tumor-bearing dogs compared with control dogs (Figures 3A,B). In the tumor size model, visual inspection of the metabolite intensity plots indicated that three control dogs (IDs 624, 627, 652) exhibited levels of Unknown 2, Unknown 3, and hippurate that overlapped with those of dogs in the large-tumor group (Figure 3A). Similarly, in the tumor malignancy model, two control dogs (IDs 032 and 627) showed intensity values for Unknown 1 and Unknown 5 within the range observed for dogs with malignant tumors. These observations were limited to visualization of the data and were not statistically evaluated. Heatmaps of the FDR-significant features further illustrated these patterns, showing a general separation between control dogs and dogs with large or malignant tumors, while separation among intermediate tumor size or malignancy groups was less distinct (Figure 3B).

Pairwise contrasts among dogs with different tumor sizes showed that four metabolite features remained significantly different between the small-tumor and large-tumor groups after covariate adjustment, of which two (Unknown 2 and Unknown 3) were also significant in the control versus large tumor comparison (Supplementary Table S3). All feature levels were found to be lower in the large tumor group (Supplementary Figure S4). However, no other pairwise contrast between groups of dogs with mammary tumors revealed features that were significant after covariate adjustment (Supplementary Figure S4).

Among features that passed the FDR threshold in the tumor size and tumor malignancy model, SIRIUS analysis of chromatographic retention and tandem mass spectra produced putative molecular identifications or subclass assignments (Table 4). MS/MS spectra for the main unknown features are provided in Supplementary Figure S5.

**Table 4:** Results from the per-contrast covariate-adjusted linear models for the tumor size and tumor malignancy.

Putative metabolite identification/molecular subclass	Putative empirical formula	Sirius score	Assumed adduct	Analytical mode	RT (min)	Mz	ID level	Logistic regression FDR	Linear model FDR	OR (95% CI)	Contrast: control vs
Unknown 6/phenylpropanoids and polyketides	C11H11NO7S	99.91%	[M-H]-	RP -	3.17	300.02	3	4,37E-02	4.30E-02	0.20 (0.048–0.79)	Malignant
Unknown 5/α amino acid and derivatives	C11H13NO7S	100%	[M-H]-	RP -	2.53	302.04	2	4,95E-02	4.30E-02	0.29 (0.093–0.93)	Malignant
Unknown 4/alpha amino acids and derivatives	C6H13N5O5S	92.47%	[M + Cl]-	HILIC -	3.18	302.03	3	4,37E-02	4.30E-02	0.27 (0.093–0.80)	Malignant
Unknown 1/benzenesulfonic acids and derivatives	C21H12N7O8S	58.33%	[M-H]-	HILIC -	1.14	522.05	4	7,14E-02	4.30E-02	0.43 (0.17–1.08)	Malignant
Hippurate/N-acyl amino acids	C9H9NO3	100%	[M + H]+	RP +	3.52	180.07	1	1,73E-01	3.00E-03	0.24 (0.041–1.43)	Large tumor
Unknown 3/alpha amino acids and derivatives	C11H22N2O9S	96.87%	[M + K]+	RP +	3.52	397.07	3	6,27E-01	9.00E-03	0.77 (0.27–2.22)	Large tumor
Unknown 2/benzenesulfonamides	C17H17ClN2O4S2	100%	[M + H]+	RP +	3.52	413.04	3	1,73E-01	3.61E-06	0.29 (0.060–1.43)	Large tumor
Unknown 1/benzenesulfonic acids and derivatives	C21H12N7O8S	58.33%	[M-H]-	HILIC -	1.14	522.05	4	1,73E-01	1.10E-06	0.26 (0.060–1.15)	Large tumor

RT, Retention time, Mz, mass to charge, ID level, Putative identification level, FDR, False Discovery Rate, OR, Odds ratio, CI, Confidence interval.

Adjusted-significant features from the covariate-adjusted linear models were further summarized in supplementary control-referenced logistic regression analyses as odds ratios per 1 SD increase in log₁₀ intensity. The logistic models were directionally concordant with the linear-model estimates for all eight FDR-significant features, with the strongest agreement observed for the tumor-size model. Some unidentified features did not remain significant in the logistic analyses, including Unknown 1 in the control-versus-malignant contrast (logistic FDR 0.07; linear-model FDR 0.043), although effect directions were unchanged. Full supplementary logistic regression results, including odds ratios, 95% confidence intervals, logistic-regression significance, and the corresponding FDR-adjusted significance from the HC3-based linear models, are provided in Supplementary Table S4.

Model diagnostics supported the robustness of the covariate-adjusted linear-model results. The main findings were consistent in robust-regression (M-estimation) and Freedman–Lane permutation sensitivity analyses, with concordant effect directions and similar ranking of the top features. Influence diagnostics based on leverage and Cook’s distance did not identify any samples that were repeatedly influential across feature-wise models. At the dataset level, Cook’s distance was modest: the highest per-subject proportion of flagged feature models was 0.33% (13/3,925 features) in Model 2. No subjects were excluded on the basis of Cook’s distance alone. Full model-diagnostics results are provided in Supplementary File 3.

Because the large-tumor group was small (*n* = 6), we further evaluated the control-versus-large tumor findings using a leave-one-out sensitivity analysis. Effect directions remained negative for all key tumor-size-associated features in all six leave-one-out iterations. Unknown 1 and Unknown 2 remained FDR-significant in all six iterations, whereas Hippurate and Unknown 3 remained FDR-significant in five of six iterations. For the latter two features, omission of dog 220 increased the FDR-adjusted *q*-value marginally above 0.05, although raw *p*-values remained <0.05 and effect directions were unchanged. These results support the directionality of the large-tumor signal but indicate that FDR-level significance for some features remains sensitive to individual large-tumor samples.

**Figure 4:**
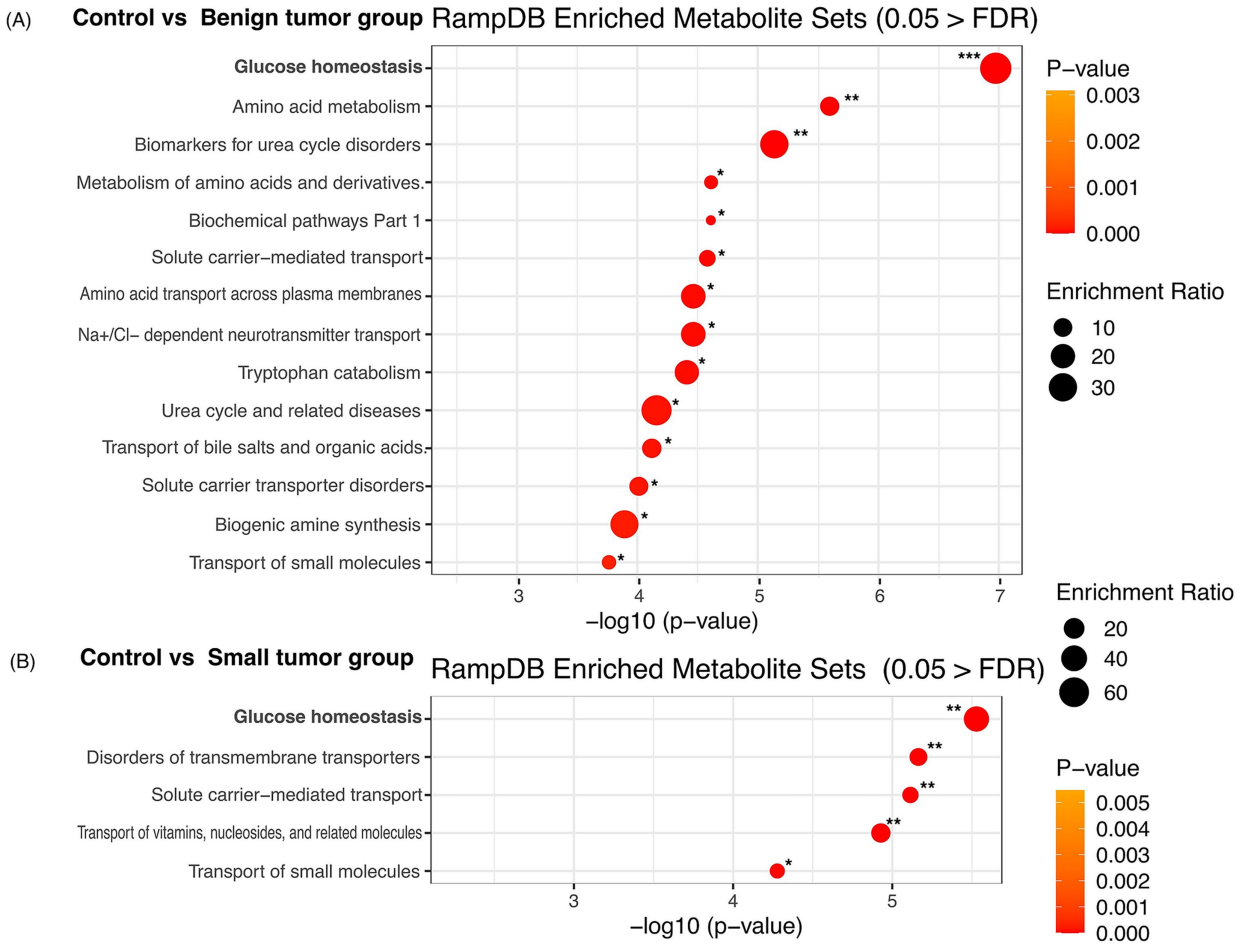
Metabolite set enrichment analysis (MSEA) of covariate-adjusted per-contrast results. Pathway enrichment was evaluated in MetaboAnalyst using RaMP-DB (Relational Database of Metabolomic Pathways). **(A)** Control versus benign tumors. **(B)** Control versus small tumors. Dot color represents the pathway p-value, and dot size represents the enrichment ratio for the corresponding metabolic pathway. Glucose homeostasis showed the strongest exploratory enrichment signal among the evaluated pathways but did not remain significant after false discovery rate correction.

Exploratory MSEA was performed using all level 1 and 2 annotated features with nominal raw *p* < 0.05 in the covariate-adjusted per-contrast linear models. Among the evaluated pathways, glucose homeostasis showed the strongest exploratory enrichment signal between control dogs and tumor-bearing groups (Figure 4). An overview of the metabolite sets included in the MSEA is provided in Supplementary Table S5, and the full metabolite sets are available in Supplementary File 4. However, glucose homeostasis did not remain significant after false discovery rate correction in the control-versus-malignant model (FDR = 0.094) or the control-versus-single-tumor model (FDR = 0.235).

## 4 Discussion

The present study identified a limited urinary metabolomic signature associated primarily with tumor size, whereas the overall adjusted case–control comparison did not yield false discovery rate-significant features. The analysis of the urinary metabolome, as a non-invasive biological matrix, offers a valuable perspective on systemic metabolic alterations associated with cancer, complementing findings obtained from tissue and serum analyses. The most robust and consistently supported finding was a reduction in hippurate concentrations in dogs with large tumors.

Hippurate is a well-characterized metabolite generated through gut microbial degradation of aromatic compounds, e.g., plant-based phenolics, or phenylalanine, which break down into benzoic acid followed by hepatic glycine conjugation (36). Decreased urinary hippurate has been repeatedly reported in human oncology studies, including those of breast (37), colorectal (38–40), bladder (41), hepatic (42, 43), and pancreatic cancers (44), where it is interpreted as a marker of systemic metabolic disturbance, altered gut microbial activity, or reduced hepatic conjugation capacity rather than of tumor-specific metabolism (45–47). Our findings in dogs are consistent with reports from human oncology studies and support the view that canine mammary tumors may reflect some shared systemic metabolic responses.

We observed that hippurate levels correlated strongly with tumor size, but not with histological malignancy or the number of mammary tumors. This suggests that tumor burden may exert a greater influence on systemic metabolism than malignancy potential. Larger tumors may increase host metabolic demand, inflammatory signaling, and amino-acid utilization, potentially reducing substrate availability for benzoate glycine-conjugation or altering gut microbial aromatic metabolism. Similar relationships between tumor volume and systemic metabolic dysfunction have been described in human cancers (48), where whole-body metabolic status often aligns more strongly with tumor burden than histologic subtype. Thus, reduced hippurate in urine may reflect the metabolic consequences of tumor load rather than intrinsic tumor aggressiveness. Local imaging approaches, such as shear wave elastography, have also been investigated for assessing canine mammary tumor tissue characteristics, particularly tissue stiffness/elasticity (49). In benign canine mammary tumors, pharmacological reduction in tumor size did not alter SWE-measured tissue stiffness, suggesting that tumor size and local tissue stiffness may capture partly distinct tumor properties. In contrast, the urinary metabolomic findings observed here are more likely to reflect systemic host-level responses associated with tumor burden rather than local tissue stiffness directly.

In addition to hippurate, several structurally unassigned, but likely aromatic and sulfur-containing metabolites showed strong covariation with both hippurate concentration and tumor size. The behavior of these metabolites was consistent among tumor-bearing dogs, and a small subset of control dogs with unusually low hippurate exhibited similarly low intensities for the same unknown features. These atypical metabolite profiles in nominal control dogs may reflect normal biological variation, but undiagnosed subclinical disease cannot be excluded. This pattern was present in both adjusted and unadjusted models. The empirical formulas of these metabolites suggest aromatic conjugates or phase-II biotransformation products, i.e., products that are covalently attached to endogenous polar groups by enzymes (e.g., glucuronide, sulfate, glutathione, glycine) to increase hydrophilicity, which facilitates renal or biliary elimination (50). Their coordinated behavior with hippurate supports the possibility that these compounds arise from systemic host processes, such as hepatic conjugation, xenobiotic metabolism, or microbial aromatic metabolism, rather than from direct tumor secretion. However, because several features remain structurally unassigned, exogenous sources such as medications, supplements, preservatives, or diet-derived compounds cannot be fully excluded.

Exploratory pathway-level analysis identified glucose homeostasis as the strongest enrichment signal in adjusted models, although this pathway did not remain significant after false discovery rate correction. This result should therefore be interpreted as a hypothesis-generating trend rather than as evidence of a confirmed pathway association. Nevertheless, the direction of this signal is biologically plausible, as altered glucose utilization, insulin signaling, and energy metabolism have been reported in canine and human mammary tumor biology. In the present study, the glucose-homeostasis signal may reflect systemic metabolic effects related to tumor presence or tumor size, but whether such alterations precede tumor formation or arise as a consequence of tumor development cannot be determined from this dataset.

Previous tissue-based metabolomic and lipidomic studies of CMTs have reported altered lipid profiles in tumor tissue, including changes in phospholipids, sphingolipids, triglycerides, and fatty-acid-related pathways (19–21). These findings support the broader view that mammary tumors are accompanied by metabolic remodeling at the tissue level. Our urine-based results do not directly measure tumor-tissue metabolism, but they are consistent with the idea that tumor-associated metabolic changes can also be reflected systemically. The urinary features observed here should therefore be interpreted as candidate markers of host-level metabolic response rather than as direct readouts of tumor-tissue lipid metabolism.

Adjustment for diet, age, sterilization status, and body size substantially reduced the number of significant metabolites compared with the unadjusted analyses. This underscores the importance of accounting for major sources of physiological variation in veterinary metabolomics. The persistence of only a small set of metabolites after adjustment, including hippurate and several unknown features, suggests that these associations are relatively robust and less likely to be driven by confounding. More broadly, these findings support the value of collecting detailed background data in comparative oncology studies, particularly when evaluating urine, which is sensitive to environmental factors, such as diet.

Notably, only four urinary metabolites distinguished malignant from benign tumors, of which two also differed between the control group and the large tumor group. Intensities of the two features, Unknowns 2 and 3, also differed between small and large tumors. These findings are consistent with the expectation that urine captures systemic metabolic alterations more readily than localized tumor histopathology. Both previous canine studies exhibited similar limitations, where the NMR dataset by Lee et al. primarily cataloged metabolite abundance without demonstrating discrimination between malignancy groups (6), and the targeted UHPLC study by Valko-Rokytovská et al. reported elevated tryptophan and tyrosine pathway metabolites in tumor-bearing dogs but did not differentiate tumor burden or grade (7). Taken together, these findings indicate that while urine is a useful matrix for detecting tumor-associated systemic metabolic changes, it may be less suitable for detecting fine-grained histological differences among mammary tumor subtypes.

## 5 Strengths and limitations

This study has several strengths. To our knowledge, this study is one of few urine metabolomics studies in canine mammary tumors and the first to incorporate diet as an explicit covariate. The study used home-living client-owned dogs, which increases clinical relevance, and combined detailed background phenotyping with histopathological classification and untargeted LC–MS profiling. Urine was collected before tumor excision, and analytical quality was supported by pooled QC samples, complementary chromatographic modes, and agreement between NMR-quantified creatinine and the corresponding LC–MS feature. In addition, the main findings were evaluated using covariate-adjusted models, robust standard errors, and supplementary sensitivity analyses.

Our study also has limitations. The case–control design relied partly on retrospective owner-reported background information, which introduces the possibility of recall error, particularly for diet and lifestyle variables. Cases and controls differed in age and other baseline characteristics, and balance diagnostics confirmed substantial imbalance and limited covariate overlap for some strata. Although these variables were included as covariates and the age-overlap restricted sensitivity analysis did not change the null adjusted case–control result, residual confounding cannot be excluded. Histopathological classification was based on routine diagnostic reports, which reflects clinical practice but limited the possibility of standardized retrospective grading. Histology slides and paraffin blocks were not available for re-review, and histological grade was not reported consistently. Therefore, the Nottingham Prognostic Index could not be applied and more detailed prognostic stratification was not possible. Tumor size was available for all cases, but its recording was not fully standardized across sources; pathology-report values were preferred, and owner-reported estimates were used only when no pathology-report measurement was available. Therefore, tumor size was analyzed using clinically defined categories rather than as a continuous variable. All control dogs underwent veterinary mammary palpation at enrollment and had no owner-reported history of mammary tumors, but imaging or histopathological confirmation was not performed in clinically tumor-free dogs. Therefore, occult non-palpable or subclinical mammary lesions cannot be completely excluded. Medication and supplement use was reported by some owners, but timing, dose, frequency, and relevance to the urine sampling period were not recorded in a sufficiently standardized manner to support covariate adjustment or sensitivity analyses. Therefore, medication- or supplement-derived contributions to unknown urinary features cannot be excluded. Breed-specific effects were not evaluated. The study included a heterogeneous client-owned dog population, and breed information was available as broad breed-class groupings rather than as a sufficiently powered breed-level variable. Because many individual breeds were represented by few dogs, breed was not included as a separate model covariate. Dog size class was included in the models, but residual breed- or breed-class-related effects cannot be excluded. Finally, although the statistical models were designed to account for the complex covariate structure of the dataset, some subgroup analyses were based on modest sample sizes and should therefore be interpreted cautiously.

## 6 Conclusion

Overall, the results of this study identify hippurate and a small number of covarying unknown conjugated metabolites (Unknowns 1–3) as candidate urinary features associated with tumor size, and a small number of unknown metabolites (Unknowns 1, 4–6) as candidate urinary features associated with malignancy status. These metabolites may reflect interactions among the tumor, the gastrointestinal microbiome, and hepatic detoxification pathways. Although they were not sufficiently specific for diagnostic use in the present dataset, they may help characterize systemic metabolic alterations associated with canine mammary tumors. Future studies integrating urine metabolomics with serum profiling, tumor tissue and/or blood gene-expression, microbiome characterization, and tumor tissue mass spectrometry imaging may help clarify the mechanisms underlying these metabolic alterations and determine their potential value in disease monitoring and biological characterization of canine mammary tumors.

## Data Availability

The raw data supporting the conclusions of this article will be made available by the authors, without undue reservation.

## Footnotes

1
https://github.com/rm00r3/canine_mammary_tumors_urine_metabolomics
