# Supplementary material for: Urinary metabolite profiles differ by tumor size and malignancy in dogs with mammary tumors
Source: Front Vet Sci. 2026 Jul 14;13:1834546. doi: 10.3389/fvets.2026.1834546 (PMC13407207; doi:10.3389/fvets.2026.1834546)
Supplement: Supplementary file 1 [file Table_1.DOCX]

Supplementary Material

# Supplementary Data

The supplementary material includes supplementary tables (Section 2), supplementary figures (Section 3), and supplementary methods (Section 4).

# Supplementary Tables:

Pathology report-based tumor diagnoses are available in Supplementary file 1.

Full results from all linear models 1-4 are available in Supplementary file 2.

Full model diagnostics for linear models 1-4 are available in Supplementary file 3.

Full metabolite sets used for the MSEA are available in Supplementary file 4.

The SIRIUS parameters are available in Supplementary file 5.

The case-control balance analyses are available in Supplementary file 6.

The leave-one-out sensitivity analyses for the large tumor group are available in Supplementary file 7.

Table S1. Overview of the per-contrast (control-versus-group) linear-model results, showing the effect of covariate adjustment on false discovery rate (FDR)-significant findings across Models 1–4.

| **Models 1-4 control contrasts: Control (n=47) vs** | **Significant features when unadjusted (FDR<0.05)** | **Significant features when covariate-adjusted (FDR<0.05)** |
| --- | --- | --- |
| Case (n=71) | 829 | 0 |
| Benign tumor (n= 29) | 242 | 0 |
| Malignant and Benign tumor (n= 16) | 226 | 0 |
| Malignant tumor (n=27) | 138 | 4 |
| Small tumor (n=53) | 647 | 0 |
| Medium tumor (n= 12) | 15 | 0 |
| Large tumor (n= 6) | 209 | 4 |
| Single tumor (n= 44) | 272 | 0 |
| Multiple tumors (n=27) | 377 | 0 |

Table S2. Significant features from the analysis of variance (ANOVA) of the tumor size model (Model 3).

| **Metabolite feature** | **Small tumor vs Control (FC)** | **Medium tumor vs Control (FC)** | **Large tumor vs Control (FC)** | **Average Expression** | **F-statistic** | **P-value** | **Adjusted P-Value (FDR)** |
| --- | --- | --- | --- | --- | --- | --- | --- |
| **Unknown 1 (HILIC_neg, m/z 522.05 @ 1.14 min)** | -2.17E+00 | -3.82E+00 | -5.41E+00 | 2.47E+00 | 1.18E+01 | 1.05E-06 | 4.14E-03 |
| **Unknown 7 (HILIC_pos50-700, m/z 297.12 @ 1.40 min)** | -6.10E-02 | 6.53E-02 | -2.37E+00 | 5.70E+00 | 1.10E+01 | 2.63E-06 | 5.16E-03 |
| **Unknown 2 (RP_pos, m/z 413.04 @ 3.52 min)** | -4.10E-02 | -8.19E-02 | -8.24E-01 | 7.49E+00 | 8.99E+00 | 2.42E-05 | 3.16E-02 |

FC: Fold change, FDR: False Discovery Rate

Table S3. FDR-significant results for covariate-adjusted pairwise disease-group contrasts in the tumor-size model.

| **Small tumor vs large tumor** | **logFC** | **AveExpr** | **t** | **P-Value** | **Adjusted P-Value (FDR)** |
| --- | --- | --- | --- | --- | --- |
| **Unknown 3 (RP_pos, m/z 397.07 @ 3.52 min)** | -2.31E+00 | 5.70E+00 | -5.40E+00 | 4.28E-07 | **1.68E-03** |
| **Unknown 2 (RP_pos, m/z 413.04 @ 3.52 min)** | -7.83E-01 | 7.49E+00 | -4.90E+00 | 3.54E-06 | **6.96E-03** |
| **Unknown 8 (HILIC_pos50-700, m/z 297.12 @ 1.40 min)** | -3.33E+00 | 5.22E+00 | -4.76E+00 | 6.43E-06 | **8.41E-03** |
| **Unknown 7 (HILIC_pos50-700, m/z 181.10 @ 1.43 min)** | -9.73E-01 | 6.51E+00 | -4.58E+00 | 1.32E-05 | **1.30E-02** |

Table S4. Full control-referenced logistic regression results for adjusted-significant features, including odds ratios (ORs), 95% confidence intervals (CIs), logistic-regression p-values and FDR-adjusted q-values, together with the corresponding FDR-adjusted q-values from the HC3-based linear models.

| **Feature** | **Contrast** | **OR (95%CI) confidence interval** | **OR_low** | **OR_high** | **p** | **q_or** | **q_lm** | **se_source** | **sd_case** | **sd_ctrl** |
| --- | --- | --- | --- | --- | --- | --- | --- | --- | --- | --- |
| **Unknown 6** | 4_Malignant (n=27)_vs_1_Control (n=47) | 0.20 (0.05-0.79) | 4.83E-02 | 7.89E-01 | 2.18E-02 | 4.37E-02 | 4.29E-02 | brglm2 | 1.08E+00 | 6.47E-01 |
| **Unknown 5** | 4_Malignant (n=27)_vs_1_Control (n=47) | 0.29 (0.09-0.93) | 9.30E-02 | 9.30E-01 | 3.71E-02 | 4.95E-02 | 4.29E-02 | brglm2 | 4.81E-01 | 9.25E-01 |
| **Unknown 4** | 4_Malignant (n=27)_vs_1_Control (n=47) | 0.27 (0.09-0.80) | 9.29E-02 | 7.98E-01 | 1.77E-02 | 4.37E-02 | 4.29E-02 | brglm2 | 6.98E-01 | 8.54E-01 |
| **Unknown 1** | 4_Malignant (n=27)_vs_1_Control (n=47) | 0.43 (0.17-1.08) | 1.71E-01 | 1.08E+00 | 7.14E-02 | 7.14E-02 | 4.29E-02 | brglm2 | 9.95E-01 | 7.18E-01 |
| **Hippurate** | 4. Large tumor (n=6)_vs_1. No tumor (n=47) | 0.24 (0.04-1.43) | 4.07E-02 | 1.43E+00 | 1.17E-01 | 1.73E-01 | 2.68E-03 | brglm2 | 3.89E-01 | 8.27E-01 |
| **Unknown 3** | 4. Large tumor (n=6)_vs_1. No tumor (n=47) | 0.77 (0.27-2.22) | 2.66E-01 | 2.22E+00 | 6.27E-01 | 6.27E-01 | 8.98E-03 | brglm2 | 2.84E-01 | 8.69E-01 |
| **Unknown 2** | 4. Large tumor (n=6)_vs_1. No tumor (n=47) | 0.29 (0.06-1.43) | 6.02E-02 | 1.43E+00 | 1.30E-01 | 1.73E-01 | 3.61E-06 | brglm2 | 4.12E-01 | 7.50E-01 |
| **Unknown 1** | 4. Large tumor (n=6)_vs_1. No tumor (n=47) | 0.26 (0.06-1.15) | 5.93E-02 | 1.15E+00 | 7.50E-02 | 1.73E-01 | 1.10E-06 | brglm2 | 2.07E-01 | 7.71E-01 |

Table S5. Overview of metabolite sets used in the metabolite set enrichment analysis (MSEA).

| **Pathway analyses from Control vs** | **Features eligible for pathway analyses Covariate adjusted raw p<0.05** | **Features matched with databases and used for pathway analyses** |
| --- | --- | --- |
| Case (n=71) | 50 | 40 |
| Benign tumor (n= 29) | 52 | 49 |
| Malignant and Benign tumor (n= 16) | 41 | 24 |
| Malignant tumor (n=27) | 47 | 41 |
| Small tumor (n=53) | 64 | 46 |
| Medium tumor (n= 12) | 18 | 11 |
| Large tumor (n= 6) | 62 | 44 |
| Single tumor (n= 44) | 50 | 36 |
| Multiple tumors (n=27) | 39 | 30 |

# Supplementary Figures

## NMR-based creatinine


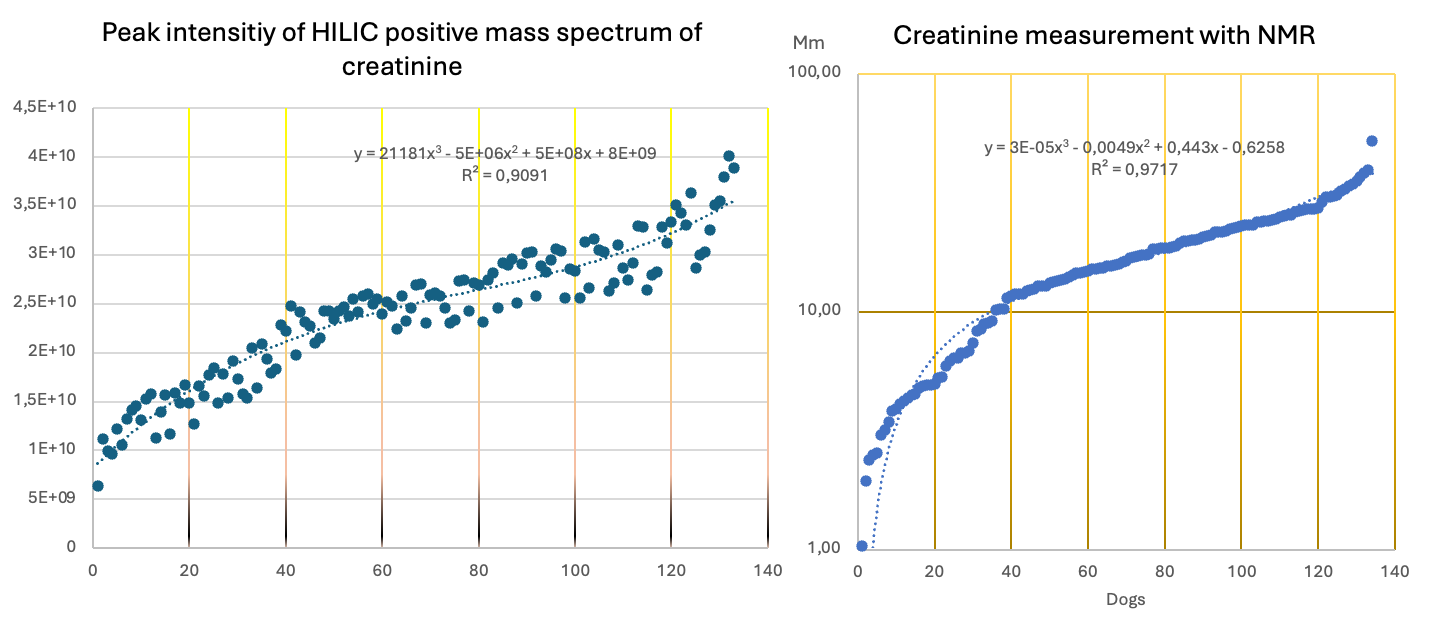


**Supplementary Figure S1.** Agreement between urine creatinine quantified by nuclear magnetic resonance (NMR) and the level 1-annotated creatinine feature detected by hydrophilic interaction chromatography–tandem mass spectrometry (HILIC–MS/MS) in the additional low-mass-range run (m/z 50–700).

## Metadata overview


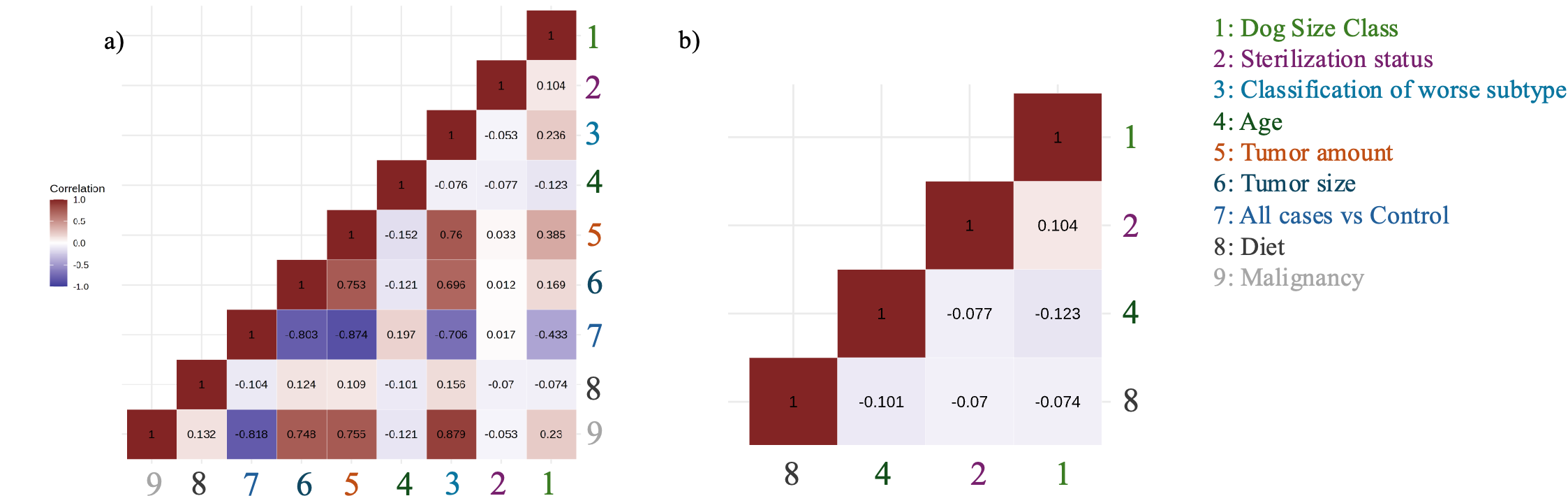


**Supplementary Figure S2.** Metadata overview. (A) Correlation matrix of study covariates measured using Pearson correlation coefficients (r), where −1 indicates maximum negative correlation and 1 indicates perfect positive correlation. (B) Visualization of the background variables included in the statistical models.

## Data distribution and quality diagnostics (data normality)


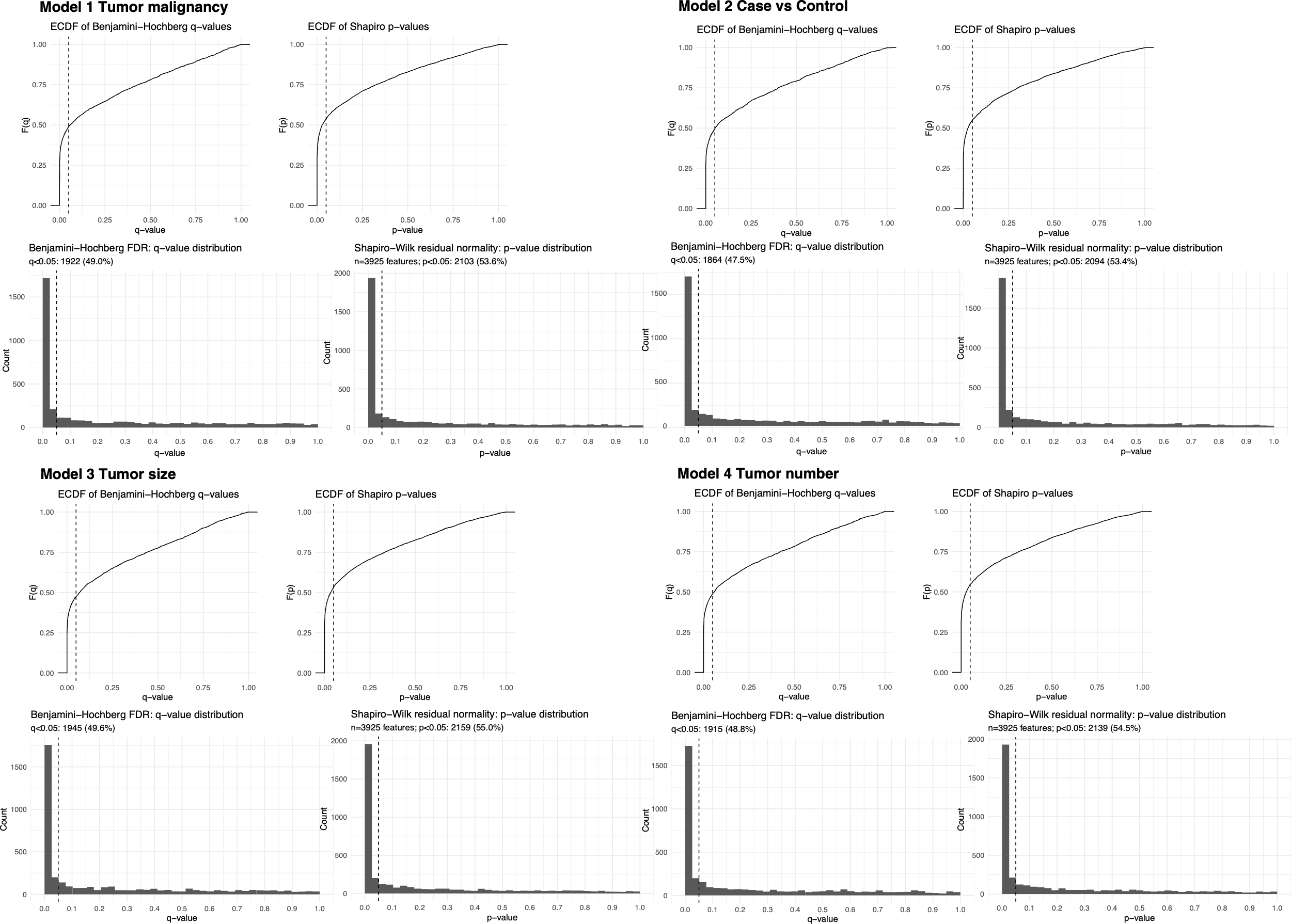


S3a

**Supplementary Figure S3.** Residual normality diagnostics for the covariate-adjusted linear models. **S3a)** Histograms and empirical cumulative distribution plots of feature-wise Shapiro–Wilk p-values and Benjamini–Hochberg-adjusted q-values calculated from residuals of the adjusted linear models. **S3b)** Representative residual Q–Q plots. **S3c)** Representative residual density plots. Representative features were selected from the distribution of Shapiro–Wilk p-values to illustrate worst-fitting, median, and best-fitting residual behavior across models.


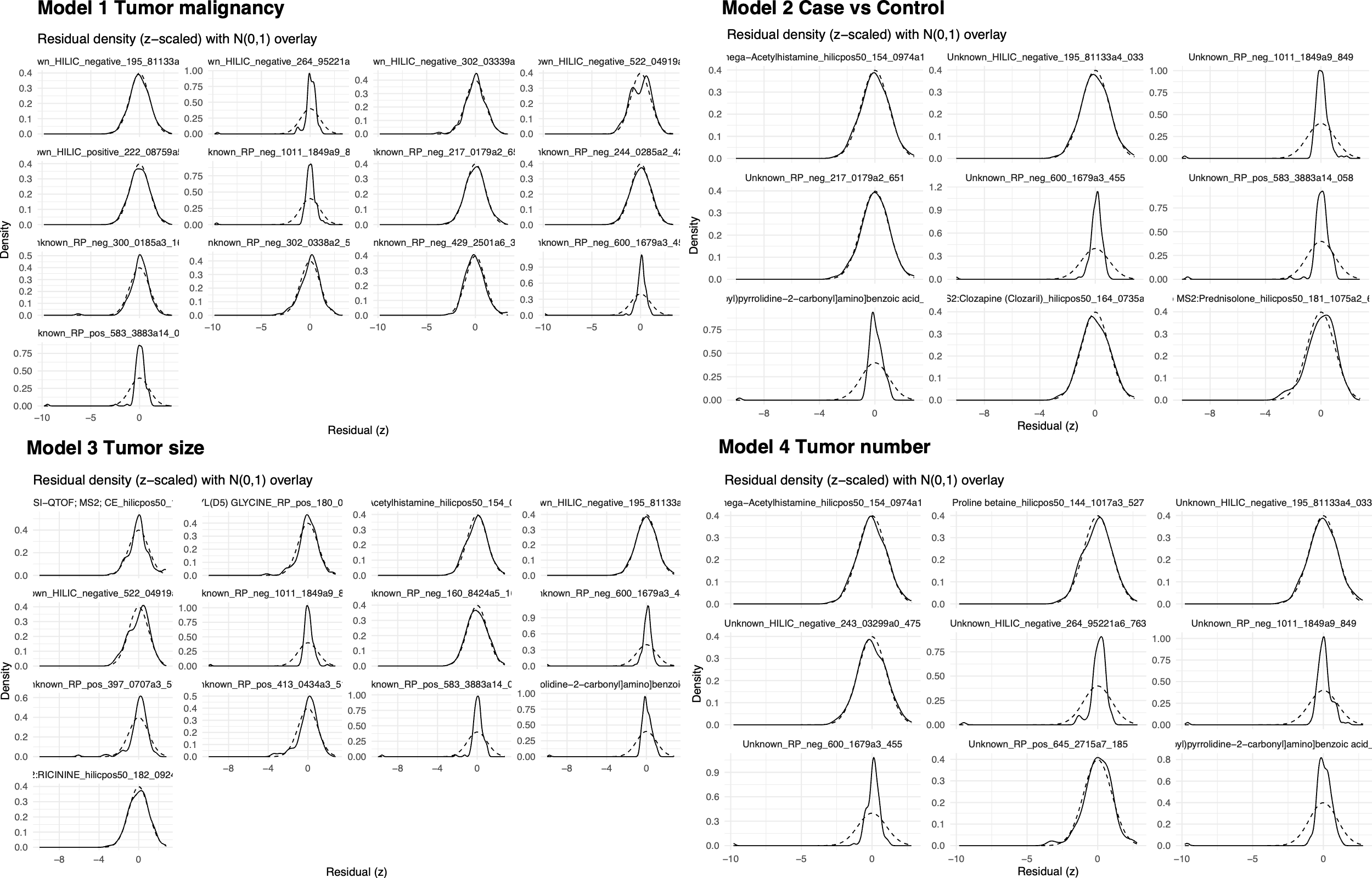


S3c


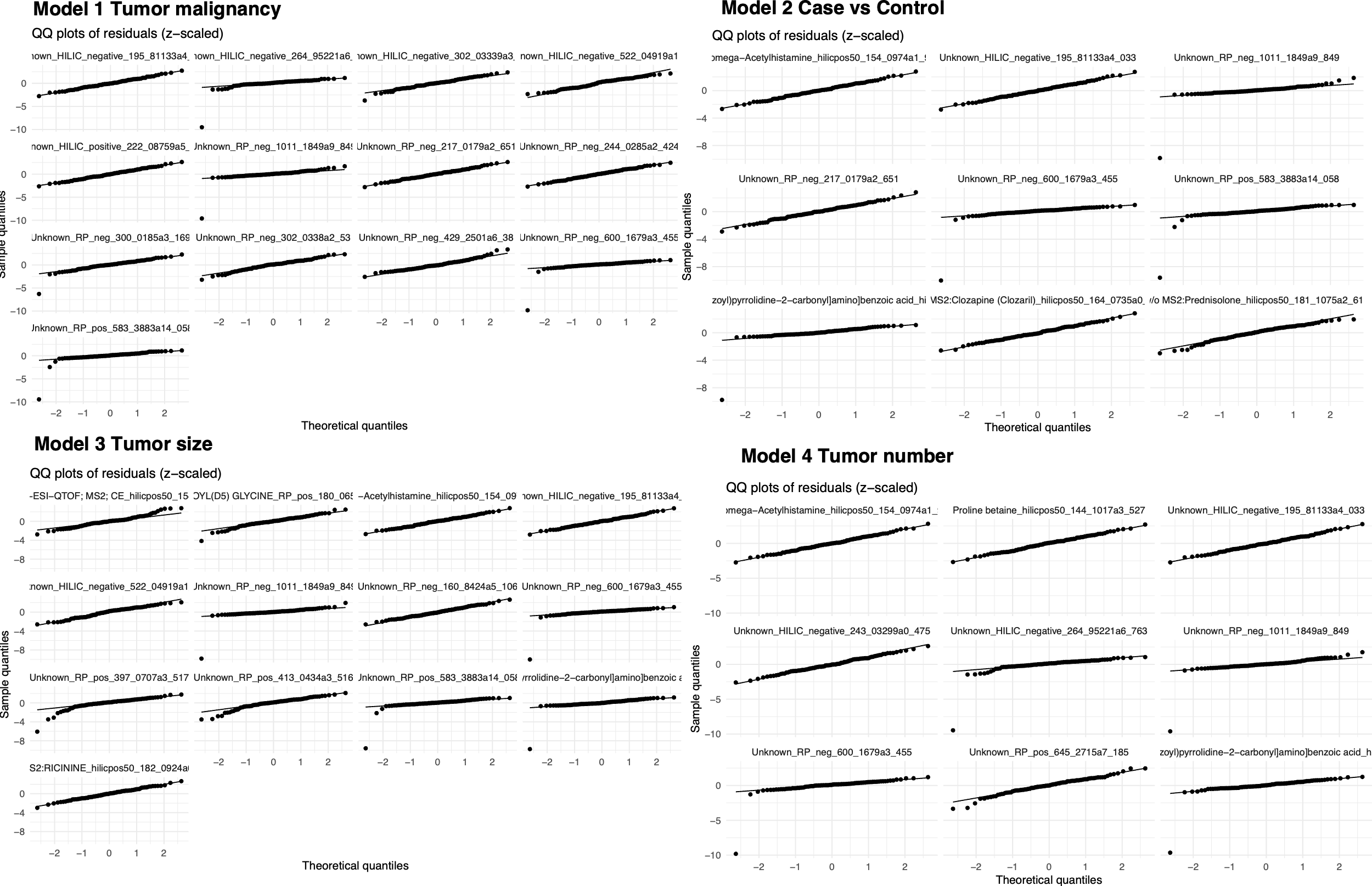


S3b

## Pairwise contrast barplots


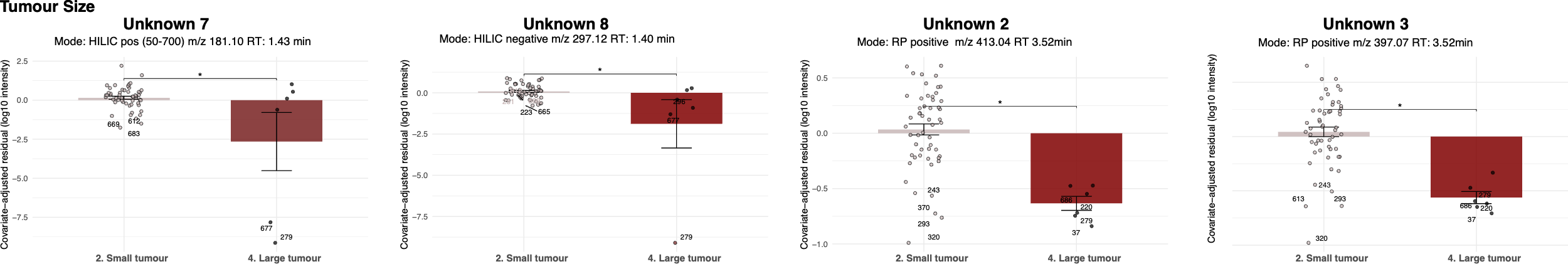


**Supplementary Figure S4.** Bar plots of features significant in covariate-adjusted pairwise tumor-size contrasts. Mean log10 intensity of the four features that remained significant after false discovery rate (FDR) correction in the pairwise comparisons among tumor-size groups.

## MS/MS spectra for the main structurally unassigned and putatively identified urinary features


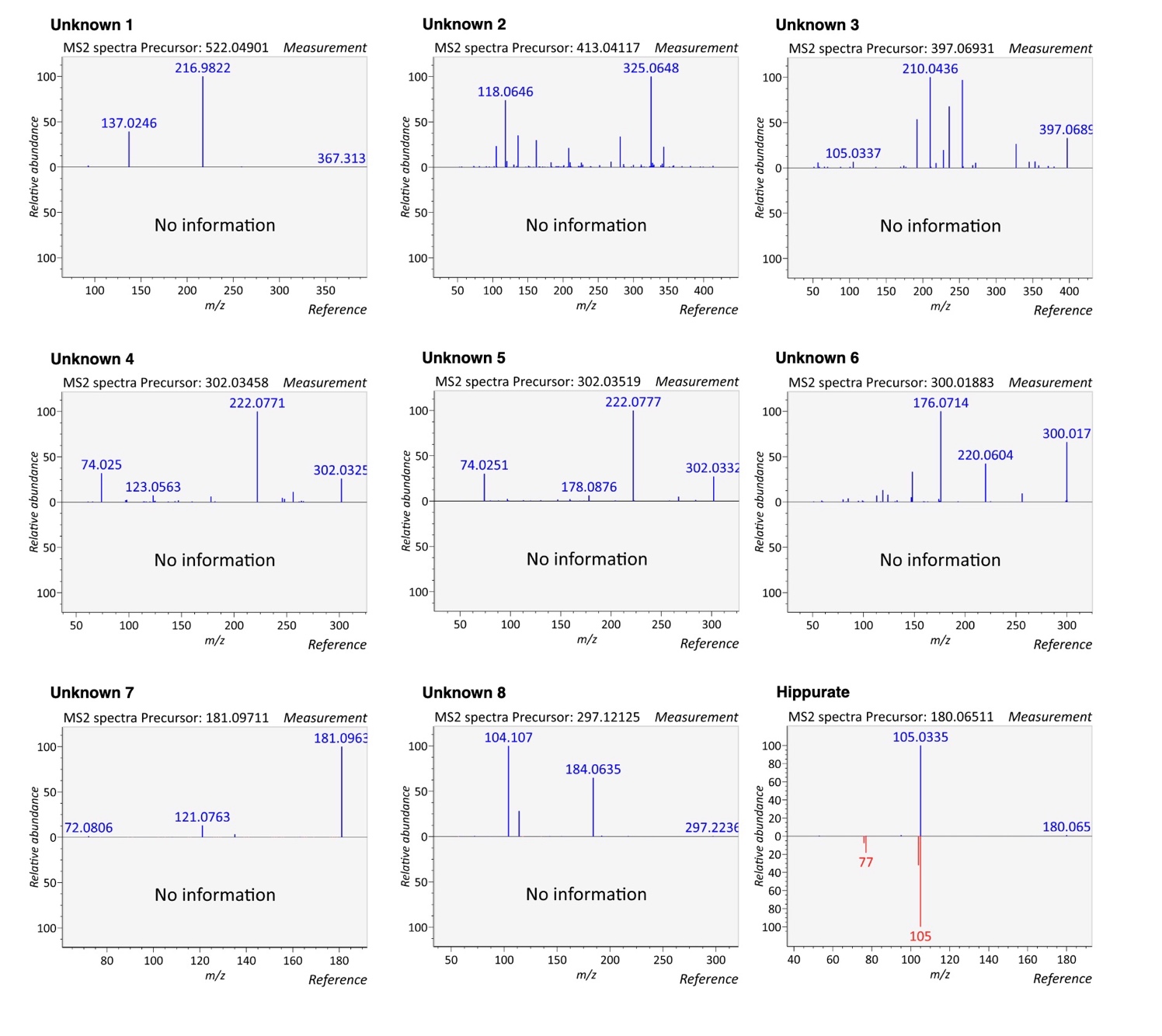


**Supplementary Figure S5.** Tandem mass spectra are shown for the eight structurally unassigned features discussed in the manuscript (Unknowns 1–8) and for hippurate/N-benzoyl glycine. For each feature, the experimental MS/MS spectrum is shown in the upper panel. When an in-house reference spectrum was available, it is shown in the lower panel for comparison; this was available for hippurate/N-benzoyl glycine only, whereas the lower reference-spectrum panels are blank for the structurally unassigned features. The unknown features should be interpreted as structurally unassigned or putatively characterized features supported by MS/MS evidence, not as confirmed metabolite identifications.

# Supplementary Methods

## Surgical removal of mammary tumors

For surgical removal of the tumors, carprofen was administered preoperatively at 4 mg/kg body weight (BW). Sedation was performed with medetomidine (0.02 mg/kg BW) and butorphanol (0.2 mg/kg BW), and anesthesia was induced with propofol (0.5–1 mg/kg BW), depending on response. Anesthesia was maintained with inhaled isoflurane (1.5%–2%). After surgery, atipamezole (Antisedan; 0.02 mg/kg BW) was administered for recovery, with the final dose adjusted according to the dog’s alertness. The surgical technique varied depending on whether the tumor had previously been diagnosed as benign or malignant. Because urine samples were collected before surgery, these surgical differences were not considered relevant to the present metabolomic analyses and are not described further here.

## Sample preparation

Urine samples were thawed in icy water and kept on ice throughout preparation. Protein precipitation/extraction used 400 µL acetonitrile (ACN; HPLC-grade, VWR Chemicals, Fontenay-sous-Bois, France) mixed with 100 µL vortexed urine and 300 µL ultrapure water (class 1, ELGA Purelab Ultra Analytical, UK). After mixing, samples were filtered using a 0.2 µm polypropylene filter plate (Agilent, Santa Clara, CA, USA) and centrifuged at 700×g for 5 min at 4 °C. QC material was prepared by pooling 10 µL from each sample, then processed identically. The filter plate was removed from the 96-well plate, which was sealed and stored at +7 °C until LC–MS analysis.

## LC–MS data acquisition

Injection order was randomized with Wranglr (github.com/antonvsdata/wranglr). Pooled QC samples were injected at the beginning and end of the batch and after every 12 study samples.

Analyses were performed on a Vanquish Flex ultra-high-performance liquid chromatography (UHPLC) system (Thermo Scientific, Bremen, Germany). Reversed-phase (RP) separations used a Zorbax Eclipse XDB C18 column (2.1 × 100 mm, 1.8 µm; Agilent Technologies, Palo Alto, CA, USA). Hydrophilic interaction liquid chromatography (HILIC) separations used an Acquity UPLC BEH Amide column (2.1 × 100 mm, 1.7 µm; Waters, Milford, MA). Injection volume was 2 µL; tray temperature 10 °C. For RP: flow 0.4 mL/min at 40 °C with water and methanol (Chromasolv LC–MS ultra, Honeywell) each containing 0.1% (v/v) formic acid (Fluka, Honeywell). Methanol gradient: 2%→100% over 10 min, hold 100% for 4.5 min, re-equilibrate to 2% over 2 min. For HILIC: flow 0.6 mL/min at 45 °C with mobile phase A (50% ACN in H₂O) and B (90% ACN in H₂O), both with 20 mM ammonium formate (Chromasolv LC–MS ultra, Fluka). Gradient (B): 100% for 2.5 min, then 100%→0% over 7.5 min, hold 0% for 2.5 min.

Ionization used a heated electrospray ionization (HESI) source on a Q Exactive Focus (Thermo Scientific). Polarity switching was performed with: spray voltage +3500 V / −3000 V; max spray current 100; auxiliary/sheath/spare gas at 10/40/2 (arbitrary units); probe heater 300 °C; capillary 300 °C; S-lens RF 50 V. Full-scan MS: m/z 120–1200 at 70,000 resolution (at m/z 200), automatic injection time, automatic gain control (AGC) target 1×10⁶. To expand small-molecule coverage, samples were additionally run in HILIC positive mode at m/z 50–700 with HESI settings: +3500 V; auxiliary/sheath/spare gas 13/50/3; probe heater 375 °C; capillary 300 °C; S-lens 50 V; automatic injection time; AGC target 1×10⁶. Data-dependent MS/MS (ddMS²): top-3 with apex triggers 0.2–3 s, dynamic exclusion 15 s; normalized collision energies 20/30/40%; resolution 17,500; isolation window 1.5 m/z; AGC target 5×10⁴. Data were acquired in Xcalibur 4.1.

## Data collection, pre-processing, and identification

All samples were analyzed in a single batch. Raw files were converted to analysis base format and processed in MS-DIAL for peak picking and alignment, following settings adapted from the NOTAME workflow. Parameters: MS¹ tolerance 0.008 Da; MS² tolerance 0.15 Da; minimum height 300,000 (RP and HILIC); minimum width 8 scans. For annotation, an in-house LC–MS/MS spectral library (University of Eastern Finland) and public databases were used for non-lipids; the built-in MS-DIAL lipid library supported lipid annotations in RP mode. Library search tolerances: MS¹ 0.008 Da; MS² 0.15 Da. Adducts followed NOTAME recommendations plus [M+ACN+H]⁺, [M+H–H₂O]⁺, and [2M+H]⁺. Alignment used retention-time tolerance 0.1 min and MS¹ tolerance 0.008 Da. Features detected in ≥20% of at least one group (N% threshold) were retained. Across five runs, 77,734 molecular features were detected; MS/MS spectra were acquired for 7,577 features. MS-DIAL flagged 828 features (low quality/poor QC detection), leaving 6,749 features, of which 1,462 matched reference spectra. Alignment results (peak areas) were exported to .xlsx for NOTAME-based drift correction and random-forest imputation of missing values per analytical mode. Identification confidence followed Sumner et al. (levels 1–4) after manual inspection of reference-matched spectra.

SIRIUS/CSI:FingerID workflow and parameters are available in Supplemental file 5.

## Statistical Model diagnostics:

Statistical model diagnostics were evaluated on the covariate-adjusted design matrix used for the main linear models. Feature-level influence diagnostics were summarized using leverage and Cook’s distance. For the strict influence screen, leverage was flagged at values greater than 3p/n, where p is the number of columns in the design matrix and n is the number of complete-case samples included in the model, and Cook’s distance was flagged at values greater than 1. Sample-level leverage summaries were obtained directly from the hat matrix of the adjusted design, and sample-level Cook’s summaries were calculated as the proportion of tested feature models in which a given sample exceeded the Cook’s distance threshold. In addition, a dataset-level Cook’s summary was generated using a feature-wise linear-model screen with a Cook’s threshold of 4/(n−p); at the subject level, dogs were flagged when at least 1% of the feature models in which they were tested exceeded that threshold. No samples were excluded on the basis of influence diagnostics alone, and diagnostic findings were interpreted together with the HC3-based main analyses and the robust-regression and Freedman–Lane permutation sensitivity analyses.

## Creatinine adjustment with NMR

Urine samples were adjusted with absolute creatinine measurements that were quantified with NMR. The quantification was performed as previously described [29]. TSP (3-(trimethylsilyl)propionic-2,2,3,3-d4 acid sodium salt) was used as an internal concentration reference.

To assess agreement between platforms, NMR-based creatinine quantification was compared with the level 1-annotated creatinine feature detected in HILIC positive mode in the additional low-mass-range run (m/z 50–700). Absolute NMR creatinine concentrations (mM) and LC–MS peak intensities were strongly correlated (R² = 0.8934; Supplementary Figure S1).
